# Linkage and association analyses of principal components in expression data

**DOI:** 10.1186/1753-6561-1-s1-s46

**Published:** 2007-12-18

**Authors:** Anthony L Hinrichs, Robert Culverhouse, Carol H Jin, Brian K Suarez

**Affiliations:** 1 Department of Psychiatry, Washington University, 660 South Euclid, Box 8134, St. Louis, Missouri 63110, USA; 2 Department of Medicine, Washington University, 660 South Euclid, Box 8005, St. Louis, Missouri 63110, USA; 3 Department of Genetics, Washington University, 660 South Euclid, Box 8134, St. Louis, Missouri 63110, USA

## Abstract

Performing linkage and association analyses on a large set of correlated data presents an interesting set of problems. In the current setting, we have 3554 expression levels from lymphoblastoid cell lines in 194 individuals from 14 three-generation Utah CEPH (Centre d'Etude du Polymorphisme Humain) pedigrees. We formed multivariate expression phenotypes from six sets of genes. These consisted of a set of genes identified by the data providers as showing common linkage to a region of chromosome 14, as well as five other sets suggested by ontological evidence. Using principal-component analyses, we generated seven quantitative phenotypes for expression levels from these six sets of genes. We performed quantitative genome linkage screens on these traits using the expression traits from the third generation of each pedigree. As expected, the strongest linkage signal was achieved when the trait under analysis was the composite of the expressions of genes previously showing linkage to chromosome 14. In particular, this trait produced a LOD score of 5.2 on chromosome 14. The trait also produced LOD scores over 3.5 on chromosomes 1, 7, 9, and 11; this suggests that these genes may be controlled by additional genetic factors on the genome. Subsequent association analyses on the first two generations of these pedigrees identified two polymorphisms on chromosome 11 as significant after correcting for multiple tests. These results suggest that principal-component analyses are useful for the analysis of pleiotropic loci. Furthermore, we have identified two single-nucleotide polymorphisms that may influence the expression of multiple genes linked to chromosome 14.

## Background

The Genetic Analysis Workshop 15 data set consists of 3554 expression levels from lymphoblastoid cell lines in 194 individuals from 14 three-generation Utah CEPH (Centre d'Etude du Polymorphisme Humain) pedigrees. In earlier work [1], the data providers performed separate linkage analyses for each expression phenotype and found evidence of pleiotropy; multiple phenotypes showed linkage to the same region on chromosome 14. Our goal was to perform analyses of composite phenotypes in hopes of identifying pleiotropic effects directly. We then followed up the strongest linkage signals with association analyses.

Principal-components analysis (PCA) is a technique for reducing multi-dimensional data sets into lower dimensions for analysis. The goal is to capture as much variation as possible of the higher dimensional data set in a lower dimensional set. Because microarray expression data typically produces thousands of correlated observations for each array, PCA is a natural analytic technique. Several other groups have shown the utility of PCA for the decomposition of expression data [2-4]; here we apply the method in the context of linkage and association analyses.

## Methods

### Principal components theory

In microarray data, PCA provides orthonormal bases for the expression array profiles and the gene transcriptional responses. The details are well described elsewhere [4].

### Linkage analyses

Our goal was to perform PCA on several different subgroups of expression phenotypes and then perform linkage analysis on the component scores. We performed the PCA in the R language [5]. In order to more closely match the sibling analysis in [1], we only used phenotypes from the sets of siblings in the third generation. In particular, we selected one sibling at random from each sibship and performed the PCA using those 14 independent individuals (assuming that the families are unrelated). Then the loadings were used to compute the components on the remaining siblings. This allowed us to perform linkage on a different set of individuals than those used for the PCA. To determine whether 14 individuals were sufficient to generate reliable components, we performed repeated samplings of 14 individuals at random from the third generation and re-generated component scores. We then examined how correlated the scores were on the overlapping individuals. We also examined the correlation with component scores generated using all 110 individuals from the third generation. Identity-by-descent matrices for linkage analysis were generated using Loki [6] on single-nucleotide polymorphisms (SNPs) thinned in order to avoid elevation from linkage disequilibrium [7]. Linkage analysis was performed using the variance-component (VC) methodology implemented in SOLAR [8].

We investigated the set of expression phenotypes linked to chromosome 14 by Morley et al. [1]. This phase consisted of 32 analyses: the expression levels of the 31 genes identified by Morley et al. [1], analyzed separately, and an analysis of the trait formed by the first component of the combined expression levels. We also examined the first component from the multivariate trait of all 3554 expression phenotypes. Finally, we used the provided gene expression data to examine PCA on three separate categories: 145 genes from the cytoskeleton category, 89 genes from the protein modification category, and 255 genes from the cell cycle category. The cytoskeleton and protein modification categories had been identified by the data providers as the most heavily represented categories among genes with highly variable expression [9]. The cell cycle category has been studied by other researchers using PCA techniques [2,3].

### Association analyses

For the strongest linkage signals, we downloaded data from the HapMap project for the CEPH families for SNPs within the two-LOD support [10]. We performed association analyses using an additive model to code the SNP genotypes using SOLAR. We also included age in the model when it was associated with the principal component. In this VC setting, age and SNP genotypes are treated as covariates predicting trait phenotype while a kinship matrix scaled by the trait heritability controls for the covariance from related individuals. We performed a two-stage analysis: first, we tested association with all HapMap non-synonymous SNPs; then, we tested association with all HapMap SNPs in the regions. We used a Bonferroni correction as well as a false-discovery rate (FDR) method (QVALUE [11]) to evaluate the significance of association in these two stages. The strategy of first focusing on non-synonymous SNPs has been applied to complex diseases [12]. Although mRNA expression levels of a single gene can be modified by polymorphisms across the gene footprint [13], in this context we are attempting to identify master regulators. We posit that non-synonymous changes may result in functional differences in proteins that regulate the mRNA expression of many other genes.

### High heritability genes

For a number of phenotypes, SOLAR reported an estimated heritability of 1.0. Further investigation revealed that the individual expression levels as well as the component scores often showed an intraclass correlation (ICC) of more that 50% in sibships. Heritability is often estimated as twice the intraclass correlation in sibships; this would imply a heritability greater than one. This is clearly nonsensical, so we suspect that this indicates a shared environment with an effect on multiple expression levels. We performed a PCA on the 471 genes that had ICC in sibships greater than 0.5 (the "high heritability" genes). We also performed further tests to determine whether these genes contained an over-representation of ontological categories. In particular, we examined the 10 most represented ontological categories in the high heritability genes, and then performed 10,000 re-samplings of 471 genes from all 3554 to determine whether any of these 10 categories were over-represented.

## Results

### Linkage analyses

We first examined the reliability of the PCA. Using the entire set of phenotypes, we found that the components generated from 14 unrelated individuals had a 98.7% correlation with the components generated from all 110 third-generation individuals. Furthermore, 10,000 repeated samplings of 14 unrelated individuals produced an average correlation of 96.1% with the 14 individuals initially chosen. We took this as evidence that the components were reliable, even using a relatively small number of individuals; we then used the same 14 individuals throughout. The PCA analysis of each of the six categories of gene expression then produced 14 principal components. Recently, Raiche et al. [14] developed a new technique to find the number of components to retain using an "acceleration factor," a measure of decrease in proportion of variance. For the cell cycle case, the analysis indicated that two components should be retained; in the other six cases, only the first component was retained. We then performed VC linkage analysis on these seven traits. We also performed linkage analysis for the separate expression traits reported as linked to chromosome 14 by Morley et al. [1] in order to replicate those findings.

The VC analysis of the separate phenotypes linked to chromosome 14 yielded fewer significant linkage peaks than originally reported; this was not unexpected because the methodology differed. In particular, the original analyses used a modification of the Haseman-Elston (HE) method [15]; under some trait models VC and HE have widely different power [16]. The VC analysis of the principal components for the seven traits revealed multiple signals in the same region of chromosome 14 as well as novel signals on other chromosomes (see Table 1). The strongest signal was of the phenotypes linked to chromosome 14, with a LOD score of 5.2 in this location. This cannot be considered as strong evidence of linkage; if the grouping of signals by Morley et al. [1] on chromosome 14 were due to chance alone, we would still expect to see a strong signal in the region. However, the linkage that appears on other chromosomes is quite interesting and would not be expected if the grouping were due to chance alone.

**Table 1:** LOD scores greater than 2.5

Principal component	Chromosome	Position	LOD score
Chromosome 14 cluster	1	30 cM	3.49
Chromosome 14 cluster	7	156 cM	3.76
Chromosome 14 cluster	9	120 cM	3.75
Chromosome 14 cluster	11	95 cM	3.73
Chromosome 14 cluster	14	81 cM	5.20
All 3554	14	88 cM	2.96
Cell cycle (first component)	3	192 cM	2.84
Cell cycle (first component)	14	86 cM	2.75
High heritability	12	118 cM	2.61

### Association analyses

For the association analysis, we focused on the five regions with linkage signals over 3.5 for the traits linked to chromosome 14, including the linked region on chromosome 14. We found the first principal component of these traits was strongly associated with age (*p *= 9.8 × 10^-6^), so we included age in all SNP analyses. We identified 761 nonsynonymous SNPs in these regions, so we set our initial significance threshold to a conservative level of 0.05/761 = 6.6 × 10^-5^. One SNP, rs10458896, is significant at this threshold (*p*-value of 4.5 × 10^-5^). The corresponding *q*-value is 0.034; this is the only *q*-value less than 0.05. This is a non-synonymous SNP in KIF18A, a kinesin family member on chromosome 11. In the analysis of all the SNPs, we found 143,798 SNPs in these regions. We set our significance threshold to 0.05/143798 = 3.5 × 10^-7^. One SNP in an intergenic region on chromosome 11, rs10768321, is significant at this threshold with a *p*-value of 5.8 × 10^-8 ^and a *q*-value of 0.008; again, this is the only *q*-value less than 0.05.

### Ontological categories of "high heritability" genes

For the analysis of the top ten ontological categories represented by the 471 "high heritability" genes, we computed empirical *p*-values based on how frequently a random selection of 471 genes contained more genes of the category in question than in the high-heritability group. We observed an over representation of the categories "nucleus" (*p *= 0.0006), "nucleotide binding" (*p *< 0.0001), "ATP binding" (*p *= 0.0001), and "RNA binding" (*p *= 0.0002).

## Discussion

As expected, PCA of traits with common linkage to a region on chromosome 14 does show a very strong signal in this region. Several other novel linkage signals appear, including two for the ontological category "cell-cycle." In principle, one would expect some genes from the same ontological category to be controlled by the same master regulators; this has been demonstrated in yeast [2]. However, as of yet, the databases in humans are incomplete and automated annotation is less accurate than manual annotation [17]. These results should be viewed with some caution. However, on the whole, these results appear to validate the use of PCA to find pleiotropic loci for multivariate phenotypes; this method has been previously shown as effective in both real [18,19] and simulated [20] data.

We also note that the association analyses were performed on a set of individuals related to, but distinct from, the individuals used in the linkage sample. In particular, some individuals from the first two generations of the Utah CEPH pedigrees have been genotyped by the HapMap project. For the linkage analyses, we used only the phenotypes of the third generation. Because the linkage analysis was restricted to a single generation, we only used age as a covariate in the association analyses. A combined linkage and association analyses would be possible if the significant SNPs, rs10458896 and rs10768321, were genotyped on the third generation of the CEPH pedigrees. We would expect that the linkage signal on chromosome 11 would be reduced in a SOLAR analysis including these SNPs as covariates if the SNPs "explain" some of the linkage signal.

The "high heritability" genes present a conundrum. We speculate that there may be shared environmental factors that influence many expression phenotypes; these factors would elevate the correlation in sibships and produce high heritability estimates. Because four ontological categories (nucleus, nucleotide binding, ATP binding, and RNA binding) are significantly over-represented in these high heritability genes, further investigation into potential environment factors modifying these functions may be of interest. These high heritability estimates do not occur when the other generations are included in the analyses; the inclusion of parents and potentially cohort effects reduce the heritability estimates.

## Conclusion

Performing PCA on a set of genes linked to chromosome 14 increases the LOD score; this cannot be considered stronger evidence of linkage because we anticipate a similar result if the clustering was due to chance. However, the linkage on chromosome 14 is supported by the analysis of all 3554 expression phenotypes where the largest peak is in the same region. We also find strong evidence for other loci on chromosomes 1, 7, 9, and 11 that control the genes linked to chromosome 14.

Association analyses were successful in identifying two polymorphisms on chromosome 11 regulating the set of genes displaying linkage to a region of chromosome 14. The association analysis was performed in two steps; first, analysis of only non-synonymous SNPs and then analysis of all SNPs in the regions of interest. The analysis of non-synonymous SNPs yielded a polymorphism on chromosome 11 in KIF18A significant after correcting for the number of non-synonymous SNPs. The analysis of all SNPs in the region yielded a SNP in an intergenic region on chromosome 11 significant after correcting for the total number of SNPs in the regions of interest. Further investigation into these SNPs may show a role in the regulation of a large number of genes.

We identified a set of genes that apparently had heritability greater than one. Shared environmental factors may increase the intraclass correlation within sibships. We found four ontological categories (nucleus, nucleotide binding, ATP binding, and RNA binding) that are significantly over-represented in these high-heritability genes; further investigation into potential environment factors modifying these functions may be of interest.

## Competing interests

The author(s) declare that they have no competing interests.
